# Beneficial Land Management for Hedgehogs (*Erinaceus europaeus*) in the United Kingdom

**DOI:** 10.3390/ani10091566

**Published:** 2020-09-03

**Authors:** Richard W. Yarnell, Carly E. Pettett

**Affiliations:** 1 School of Animal, Rural and Environmental Science, Brackenhurst Campus, Nottingham Trent University, Southwell NG25 0QF, UK; 2 Wildlife Conservation Research Unit, Department of Zoology, Recanati-Kaplan Centre, University of Oxford, Tubney House, Abingdon Road, Tubney, Oxfordshire OX13 5QL, UK; carly.pettett@gmail.com

**Keywords:** insectivore, agri-environment schemes, habitat preference, farmland biodiversity, small mammal, conservation

## Abstract

**Simple Summary:**

Hedgehogs are declining in the United Kingdom and are now absent from large areas of agriculture land. This commentary discusses the requirements of hedgehogs and links these to land management options that are currently used to benefit wildlife in agricultural areas. Using our knowledge of hedgehog requirements for population persistence, we suggest which land management practices are likely to be of benefit to hedgehogs in the hope that land owners will adopt some of the suggestions to help maintain and expand existing hedgehog populations across agricultural landscapes of the United Kingdom.

**Abstract:**

Hedgehogs (*Erinaceus europaeus*) are traditionally thought of as being a rural dwelling species, associated with rural and agricultural landscapes across Europe. However, recent studies have highlighted that hedgehogs are more likely to be found in urban than rural habitats in the United Kingdom. Here, we review the status of rural hedgehog populations across the UK and evaluate the potential benefits of agri-environment schemes for hedgehog persistence, while highlighting a lack of empirical evidence that agri-environment options will benefit hedgehog populations. Our synthesis has implications for future conservation strategies for hedgehogs and insectivorous mammals living in agricultural landscapes, and calls for more empirical studies on agri-environment options and their potential benefits to hedgehogs.

## 1. Introduction

The western European hedgehog (*E. europaeus*) (hereafter referred to as ‘hedgehog’) is a species of conservation concern in the United Kingdom [1,2,3], due to reported population declines [4,5,6]. Possible reasons for decline may include agricultural intensification leading to the loss of habitat complexity due to the removal of hedgerows and increased field sizes [7,8,9]; limited connectivity and fragmentation in rural landscapes [10]; reductions in food availability due to wide scale pesticide use and cultivation, and possible climate mediated effects [11,12]. The Eurasian badger (*Meles meles*) is also implicated in the decline of hedgehogs due to their increasing abundance and through the mechanism of intra-guild predation [13,14]. Mortality risk associated with road traffic collisions may also have increased due to an increase in the density of road networks and associated traffic [15,16,17,18,19]. Conservation actions are therefore required to identify key habitats for hedgehogs and habitat management to ensure hedgehog persistence in the wider countryside.

Hedgehogs are traditionally thought of as being a rural dwelling species, associated with Europe’s countryside and agricultural landscape. However, recent studies have highlighted that hedgehogs are more likely to be found in towns and cities [20,21,22,23], and have higher densities in urban areas [24,25] compared to agricultural landscapes.

Other studies have also struggled to find hedgehogs on agricultural land, and more often closely associated with villages surrounded by an agricultural matrix [9,20,21,23]. Indeed, hedgehogs were only found in 22% of 262 survey sites across the UK [26]. This is particularly worrying since 70% of the land across the United Kingdom is made up of rural agricultural habitats [27], suggesting that hedgehogs are no longer ubiquitous across these landscapes, but rather have a much more patchy discontinuous distribution.

Where hedgehogs do exist in the rural landscape, they display clear habitat preferences for pasture fields, and avoidance of arable and woodland habitats [26,27,28]. Arable areas may be used if bordered by field margins and extensive hedgerows as prescribed by agri-environmental schemes [8,29,30]. Furthermore, hedgehogs in southern Ireland actively selected arable fields for foraging after the autumn harvest [31]. Therefore, hedgehogs can and do persist in agricultural landscapes. However, no studies have empirically tested whether changes in agricultural land management can lead to changes in the density and distribution of hedgehogs.

Agri-environment schemes are designed to help land managers manage their land in an environmentally friendly way and many options have been suggested as being beneficial to hedgehogs. The uptake of agri-environment schemes by land managers in the UK has increased since their inception in the late 1980s [32]. In 2018, 3.2 million hectares were managed under some form of agri-environment scheme, which is 18% of the total agricultural land area of the UK [33]. Despite the increase in the uptake of agri-environment schemes, hedgehogs have continued to decline [6], which suggests that either agri-environment schemes are not being implemented across sufficient areas of land or that they are not beneficial to hedgehogs. Here, we will review literature on hedgehog ecology to provide an informed insight as to which agri-environment options are likely to benefit hedgehogs and evaluate the evidence pertaining to these.

## 2. The Importance of Food Availability

Hedgehogs require an abundant and varied macro-invertebrate prey base and any habitats that provide this spatially and temporally are likely to benefit hedgehogs. Hedgehogs are principally insectivores, but they also take a wide range of more unusual food items such as bird eggs, small mammals, amphibians, and occasionally fruit [23,34]. What a hedgehog eats on any given night will depend on local food availability which is determined by a range of factors such as the local habitat, daily weather, and temperature [34]. Therefore, a hedgehog’s diet will vary from night to night and throughout the year [35,36], suggesting that a diversity of food will be beneficial.

Food availability is often cited as having a positive influence on hedgehog distribution [37] and has been shown to influence hedgehog habitat selection [8,11,30,31]. The ability of hedgehogs to associate with food rich patches has also been demonstrated experimentally [37]. Therefore, where prey abundance is high or becomes more readily available at a site, they are likely to be exploited.

Sites of high food availability have also been used to explain variation in hedgehog density [23,24,37]. For example, supplementary feeding in villages has been suggested as the cause of observed higher hedgehog densities in villages compared to the surrounding agricultural landscape [24]. Natural prey availability may also be higher in villages due to lower pesticide application relative to agricultural landscapes [24]. Lower food availability in agricultural landscapes has been suggested as causing larger hedgehog home ranges, where individual movements need to be larger to meet daily energy requirements [38]. Therefore, it appears the daily resources required by hedgehogs are more widely distributed on agricultural land compared to villages that have higher food availability. The impacts of pesticides on hedgehog prey abundance and the concurrent role of supplementary feeding on hedgehog movement and abundance requires further investigation.

Competition for food resources may also impact hedgehogs, although no empirical studies have investigated this [34]. The degree to which competition influences population size is unknown [39], but hedgehogs have been shown to co-exist with larger competitors such as badgers and foxes (*Vulpes vulpes*) in urban areas where food resources are thought to be abundant. Abundant food resources therefore are likely to allow co-existence across a range of mammals and may allow greater niche differentiation. Individual hedgehogs also face intra-specific competition with population size regulated via density dependence [29], further supporting the idea that increases in food availability will increase population size.

If food availability is limiting hedgehog density and distribution in rural agricultural habitats [9,23], it follows that any land management that increases hedgehog prey abundance is likely to be beneficial to hedgehogs locally.

## 3. The Need for Habitat Connectivity

Habitat fragmentation is one of the major drivers of global biodiversity loss. Small areas of fragmented habitat result in small populations which face higher probabilities of extinction. Recent studies on hedgehogs suggest that their populations are highly fragmented in the rural agricultural landscape [26]. Furthermore, rural local populations are genetically differentiated [15], suggesting hedgehogs are either poor dispersers or that barriers to dispersal are fragmenting populations [40]. Therefore, identifying and creating suitable connecting habitat such as hedgerows or field margins can reduce the probability of local extinction [41].

Some of the insights into hedgehog movements come from studies that have released rehabilitated hedgehogs into the rural environment. These studies have shown that hedgehogs move quickly through agricultural landscapes, presumably in search of areas occupied by other hedgehogs [28,29,42,43]. Hedgehogs released into novel surroundings undergo highly variable exploratory movements, showing a significant preference for urban areas while avoiding agriculturally dominated areas [42,44]. Whether naturally dispersing hedgehogs follow these patterns is uncertain and further research into how and over what distances hedgehogs disperse is needed to fully inform the functionality of habitat corridors. However, based on the available evidence, it is plausible that hedgehog movement through the landscape is likely to be facilitated by greater habitat complexity, with increases in the number of linear features such as hedgerows, and smaller field sizes that would facilitate movement and improve population connectivity [41].

## 4. Shelter Resources

One of the most important resources for hedgehogs is the nest site. During the day, hedgehogs will rest in a nest which provides security and protection from the elements. The nest is also important during winter hibernation [45,46], and for giving birth to young [34]. Nests vary in construction materials and location, but typically comprise broad leaves and or grass constructed in a supporting structure of brambles or hedge [34]. In the UK, the majority of nests are sited within thorny or stinging vegetation, under bramble, holly, hawthorn, or nettles [46,47,48]. Hedgehogs will rest up in any dense vegetation and in summer, may forego the construction of nests and simply sleep in dense vegetation [34].

Winter nests for hibernation are also particularly important to hedgehogs. In the UK, hibernation usually starts around mid-November and continues through to Mid-March, but the exact timings vary due to local weather conditions, with a mean hibernation period of 149 nights in southern Ireland [12]. Most individuals will become active for short periods over winter, typically in response to warmer temperatures, and on average hedgehogs, will use four to five nests per winter [45,46]. In warmer climates such as Italy, hibernation lasts for two months between January and February [49]. Therefore, nest availability or structures that could support nests are an important resource for hedgehogs and any enhancements of such features are likely to be beneficial.

## 5. The Role of Habitat Mediating Badger Predation

Predation is a natural process that can regulate prey densities. In natural systems where prey and predator co-exist, the predation of prey does not result in the extinction of the prey due to a large number of complex interactions such as: the density of predators and prey; predators being able to predate a wide range of prey; and prey having evolved strategies to reduce individual chances of predation, such as camouflage and avoidance of areas with predators. These complex interactions work together to make it difficult to predict how the predation by one species will influence the prey populations.

Hedgehogs could be prey to a small number of predators in the UK. The main predator is the badger, but foxes and pet dogs (*Canis familiaris*) can also cause some mortality, especially in juveniles [34]. The degree to which predation could limit hedgehog populations is unknown, as are the badger predation rates experienced by hedgehogs across their distribution. However, there is evidence that hedgehogs spatially avoid badgers [20,50], and that in areas where badger numbers have been reduced via culling, local hedgehog numbers have responded positively [39].

Despite these trends and relationships, no empirical study has been able to demonstrate the mechanism by which badgers would exert a negative population response on hedgehogs, i.e., whether this is through direct predation, competition for food resources, or via a landscape of fear. Indeed, there are many areas in the UK where badgers and hedgehogs are known to co-exist [21,26], and hedgehogs have also been shown to decline in areas with low badger sett density [5]. Therefore, it seems that badgers may exert a negative influence of hedgehogs, but factors other than badgers are also having an impact on hedgehog distributions.

Whether particular features in the landscape can help hedgehogs avoid or reduce predation is unknown. It may be that at a fine scale, badgers and hedgehogs prefer different habitats across the landscape. In the Netherlands for example, the distribution of hedgehogs was positively influenced by recreational areas (parks), urban areas, and roads, whilst these factors negatively influenced the distribution of badgers [22], suggesting that either hedgehogs are spatially avoiding badgers or that both species have differing habitat preferences. Further research on each species habitat preference is needed to untangle these affects.

Where both badgers and hedgehogs co-exist, one may speculate that habitat features such as dense vegetation, intact hedgerows, and areas of scrub may help provide suitable refuges that make it harder for badgers to find and predate hedgehogs [30]. Hedgehogs do stay closer to edge habitats in areas frequented by badgers than in areas without them [30]. Under such circumstances, any increase in edge habitat such as smaller fields, increased hedgerow extent, and possible extensive field margins could facilitate hedgehog persistence in areas with badger presence.

## 6. Beneficial Management Actions

For hedgehog populations to persist in the UK’s rural countryside, management action that helps provide suitable sites for shelter, connectivity and abundant food are essential. However, as this review indicates, very few studies have empirically tested whether increases in food availability, or improvements in habitats result in hedgehog population improvements. This knowledge gap needs addressing before resources are devoted to habitat improvements specifically for hedgehogs. In the meantime, many of the options included in current agri-environment schemes designed to improve habitat for farmland birds and invertebrates could also benefit hedgehogs (Table 1). In many cases, food, connectivity, and shelter can be increased under the same management actions. For example, maintaining and increasing hedgerow density should provide nest sites, shelter, and refuge from predators while increasing invertebrate prey biomass and provide corridors for dispersal between neighboring populations [30]. Using the information provided in the review above, we contend that the following management actions are likely to be beneficial to hedgehogs, as well as wider biodiversity targeted beneficiaries such as farmland birds, and re-assure landowners that the implementation of these agri-environmental schemes is likely to have genuine biodiversity benefits.

### 6.1. Hedgerow Availability and Density

Increasing the density, width, height, and length of hedgerows on agricultural land will benefit many species [51] including hedgehogs. To be of benefit, species rich hedgerows with stands of trees will improve hedge structure increasing invertebrate abundance and diversity [52] and improve food availability. Maintaining and re-establishing well connected hedgerows with bramble understorey and good ground cover within arable habitat is recommended to enhance the suitability of fields for hedgehogs both during summer foraging and winter hibernation [12]. Species such as brambles and rose will also improve the structure of the hedge for nest sites [34]. Mature trees provide additional nesting material and are also beneficial to invertebrate prey [53]. If combined with appropriate field margins, the hedgerow matrix across the landscape will allow dispersal and movement linking hedgehog populations [41], reducing fragmentation and increasing population viability.

The size and management of hedgerows may also improve the wider landscape characteristics that are beneficial for hedgehogs. Larger hedges will provide more shelter and foraging opportunities for hedgehogs than small ones. Hedges that are over 3 m in height and flayed in winter (January onwards) on a 3-year rotation will provide robust and healthy hedges that have high flower and fruit yields [54], which has wider biodiversity benefits for many invertebrates [55] and their predators. Cutting on rotation will also ensure that two thirds of hedges will be uncut in any year, reducing the levels of mulch at the base of the hedge which can hamper vegetative growth needed for nest construction in the understorey. Ideally, the base of the hedge should be greater than 2 m wide, with dense vegetation at the base of the hedge with no gaps. When cutting hedges, care must be taken not to cut the vegetation at the base of the hedge to protect nesting and hibernating hedgehogs.

Hedges are often fenced to prevent damage from livestock, and farmers can receive payments for fencing which can ensure a good hedge for nesting, foraging, and dispersal. However, the size of fence mesh needs consideration to ensure they are large enough to allow hedgehogs to pass through and access the hedge.

### 6.2. Field Margin Availability and Management

Agri-environment schemes that (re)create hedgerows and establish field margins are recommended for hedgehog conservation, particularly in intensively farmed arable landscapes where food availability and nesting sites are sparse [11]. Of great importance is the presence of a hedge buffer or headland that will provide additional cover, nest material, and invertebrate prey. The margin of grass should be allowed to extend by at least 2 m from the base of the hedge into the crop. Where possible, wide grassy/bushy margins around agricultural fields should be established to help hedgehogs disperse and access to suitable resources [41]. Unmanaged grassy margins in pasture fields should also be encouraged to provide summer nesting/resting up areas [41]. Establishing and/or maintaining 4–6 m grassy field margins and incorporating conservation headlands in arable dominated landscapes is recommended and supported by agri-environment schemes to provide refuge and foraging habitat [30].

Tussocky grass can be used by hedgehogs for cover and daytime nests during summer, and where this is particularly thick, it may be used for hibernacula in winter. Such dense undergrowth also provides good habitat for beetles, spiders, and caterpillars [56] which are important food.

Beetle banks that cross large arable fields will improve food availability for hedgehogs and potentially act as corridors for movement. The benefits to the landowner for maintaining a beetle bank is that it will support predatory spiders and beetles that will migrate into the crop and feed on aphids and other pest species that feed on crops [57]. This will reduce pest damage and reduce the need for insecticides, providing economic benefits for the farmer [58].

### 6.3. Field Size in Relation to General Habitat Availability in the Landscape

Large field sizes are likely to hinder hedgehog movement due to their propensity for using field boundaries. Therefore, landscapes with high density of linear features and small land parcels will be advantageous for movement. Where field sizes are large, the addition of field margins, robust hedges, and features such as beetle banks will make these habitats more penetrable, while also providing additional food resources [41,43,44].

Hedgehogs are capable of long-distance dispersal (>4 km) and thus populations located within this range are unlikely to be isolated [42]. Habitat edges, particularly roads and hedgerows, are utilised as dispersal corridors by hedgehogs, thus management activities should be sensitive to the potential presence of hedgehogs and should focus on increasing suitability by maintaining connectivity between linear features [30].

Management actions that increase the diversity of invertebrate species are recommended to allow for niche partitioning amongst different age classes of hedgehogs and include but are not limited to agri-environment options that include buffer strips, establishing and managing hedgerows, organic farming, and beetle banks [36]. Having diverse land use will also increase heterogeneity in the landscape, creating habitat for a greater diversity of wildlife. As such, mixed farms with areas of pasture, arable crops, and set-aside fields have the potential to be beneficial to hedgehogs and are more likely to support a viable hedgehog population than single use arable farms [59]. Increasing heterogeneity in the landscape by increasing edge habitat, copses, different land uses with amenity, and garden habitats surrounding buildings will all help to provide a diverse array of shelter and foraging resources.

### 6.4. Cropping and Ploughing Regimes in Relation to Prey Availability

Traditional farming methods that include mosaics of pasture and arable, well connected hedgerows, over-winter stubble, and fodder crops are recommended to increase the suitability of arable land for hedgehogs [30]. The greater the diversity of land types will provide a greater array of habitats types from which to support invertebrate prey that is needed throughout the year [36]. Where possible, areas that are dominated by arable crops could enhance habitat in less economically important habitats in the wider arable landscape. For example, habitat around farm buildings could be enhanced to support local populations, with enhancement of gardens, amenity grassland, and small pasture fields that could provide refuge habitats.

Organic farming will increase prey availability and has great potential as rural hedgehog habitat due to the increased invertebrate abundance associated with organic farms [60]. Many taxa have been shown to benefit from organic farming practices through increases in abundance and species richness which are principally driven by reductions in the use of pesticides and herbicides, sympathetic management of non-cropped areas, and utilisation of mixed farming. Such practices are likely to be beneficial to hedgehogs as well and may be equally as beneficial if targeted at specific areas on non-organic farms [59].

Reduced tillage will increase earthworm abundance [61,62] and may increase arable field use by hedgehogs [31]. There is a direct negative relationship between earthworm abundance and the depth of tillage, with no-till and conservation agriculture providing highest earthworm abundance. Switching from conventional tillage will also improve levels of soil organic matter, reduce the depth of the soil organic layer, and reduce soil compaction [62]. However, it is acknowledged that switching from conventional tillage to no-till or conservation agriculture will take up to 10 years for improvements in soil structure and health and associated earthworm biomass [62].

### 6.5. Areas of Scrub and Decaying Vegetation

Unkept areas providing cover and leaf litter are often utilised by hedgehogs principally for nest building [47] but also as a habitat for invertebrate prey [11]. Leaf litter is an important nesting resource for hedgehogs throughout the winter and should be either left or collected into piles near potential hedgehog nesting sites, such as tree lines, copses, or hedgerows [48]. Sheltered areas with bramble should be established and/or maintained as these provide important hibernacula sites that have increased longevity and lower daytime temperatures which may reduce arousal and thus risk of mortality of hedgehogs over winter [48]. Management of scrub during winter should be sensitive to hibernating hedgehogs, whereby areas of scrub and piles of leaf litter should be left intact [47,48].

## 7. Conclusions

Rural hedgehog populations across the UK tend to be located in villages more than the surrounding agricultural matrix of habitats [44] and it is important for their future persistence that the wider rural landscape can be utilised by hedgehogs. Based on our understanding of hedgehog ecology, we contend that many agricultural schemes designed to combat biodiversity loss would also benefit hedgehogs including enhancements to the extent and size of hedgerows and field margins, less intensive agriculture, and more diverse farming types. These changes will likely improve food availability, shelter, connectivity, and possibly reduce predation by badgers. Unfortunately, there is a lack of conservation evidence that such changes would result in tangible benefits to hedgehogs and more research is required to test these suggestions so that more specific targeted actions for hedgehogs can be proposed and implemented. Without such action, it is likely that hedgehogs will only be found in urban habitats in the future.

## Figures and Tables

**Table 1:** Summary of agri-environment management options that benefit biodiversity and are likely to be of benefit to hedgehogs (*Erinaceus europaeus*) in agriculturally dominated landscapes in the United Kingdom.

Management Action	Key Requirements	Benefits to Other Species	Potential Benefits for Hedgehogs
Hedgerow management	Maintain a hedge at least 2 m tall and 1.5 m wide. Cut hedgerows: either no more than 1 year in 3 or no more than 1 year in 2. In-fill any length of hedge with more than 10% gaps.	Increases blossom for invertebrates, food for overwintering birds and nesting habitat.	Shelter, corridors for movement connecting populations and invertebrate food.
Field margin availability and management	Establish or maintain a 4 to 6m wide grass buffer strip. Cut between 1 and 3 m of the strip next to the crop edge every year after 15 July. Only cut the remaining width to control woody growth.	Provides habitat and movement corridors for wildlife.	Corridors for movement connecting populations and invertebrate food.
Beetle banks	Create or maintain an earth ridge, measuring between 3 m to 5 m wide and at least 0.4 m high. Establish or maintain a tussocky grass mixture.	Provides nesting and foraging habitat, benefiting invertebrate biodiversity, small mammals and barn owls	Corridors for movement connecting populations and invertebrate food.
Areas of scrub and decaying vegetation	Only cut to maintain the scrub and grass mosaic and to control the spread of noxious weeds and invasive non-native species. Protect growing trees from livestock and wild animals.	Provides enhanced habitat for wildlife such as birds and invertebrates.	Shelter, corridors for movement connecting populations and invertebrate food.
